# Treatment strategy for atypical ulnar fracture due to severely suppressed bone turnover caused by long-term bisphosphonate therapy: a case report and literature review

**DOI:** 10.1186/s12891-020-03824-y

**Published:** 2020-12-03

**Authors:** Kensaku Abe, Hiroaki Kimura, Norio Yamamoto, Shingo Shimozaki, Takashi Higuchi, Yuta Taniguchi, Takaaki Uto, Hiroyuki Tsuchiya

**Affiliations:** 1 Department of Orthopaedic Surgery, Japanese Red Cross Kanazawa Hospital, 2-251 Minma, Kanazawa, 921-8162 Japan; 2 Department of Orthopaedic Surgery, Graduate School of Medical Sciences, Kanazawa University, 13-1 Takara-machi, Kanazawa, 920-8641 Japan

**Keywords:** Atypical fracture, Ulna, Severely suppressed bone turnover, Open reduction and internal fixation, Bone graft, Dual plate, Low-intensity pulsed ultra sound, Teriparatide

## Abstract

**Background:**

Atypical fractures may occur due to the combined effect of severely suppressed bone turnover (SSBT) caused by long-term bisphosphonate treatment and chronic repetitive bone microdamage. Atypical fracture of the ulna due to SSBT is a rare entity; there is no standardized treatment strategy for this condition. We successfully treated a patient with atypical fracture of the ulna. Herein, we present this patient, review the relevant literature, and discuss the treatment strategy.

**Case presentation:**

An 84-year-old woman presented with atypical fracture of the left ulnar shaft due to SSBT. She had a history of bisphosphonate therapy (ibandronate and alendronate) since more than 10 years; her bone turnover was severely suppressed. We performed open reduction and internal fixation (ORIF) using dual plate with some additional treatments. These included drilling and decortication, use of autogenous bone graft, low-intensity pulsed ultrasound (LIPUS) treatment, and administration of teriparatide. Finally, bone union was observed at 11 months after surgery.

**Conclusions:**

Based on the literature review and our experience with this case, ORIF alone may not be adequate to achieve bone union; drilling, decortication, and use of cancellus bone graft is important to achieve favorable outcomes. Administration of teriparatide and LIPUS may facilitate early bone union, although further studies are required to provide more definitive evidence. Furthermore, ORIF using dual plate may help avoid implant failure owing to the long time required for bone union.

## Background

Atypical fractures may occur due to the combined effect of severely suppressed bone turnover (SSBT) caused by long-term bisphosphonate treatment and chronic repetitive bone microdamage [1–4]. Reports of atypical fractures in patients receiving long-term bisphosphonates have largely pertained to femoral fractures; there is a paucity of data about atypical fractures at other sites in this setting [5]. Atypical fractures of the upper extremities are rare [6]. On literature search in April 2020, we could identify only 18 reports describing 25 atypical fractures of the ulna in the English and Japanese literature (11 and 7 reports, respectively) (Additional file 1) [5–22]. In this case report, we describe the diagnosis and successful treatment of a patient with atypical fracture of the ulna. In addition, we summarize the relevant literature and discuss the treatment strategy. Written informed consent was obtained from the patient for the publication of this case report and the accompanying images.

## Case presentation

**Fig. 1:**
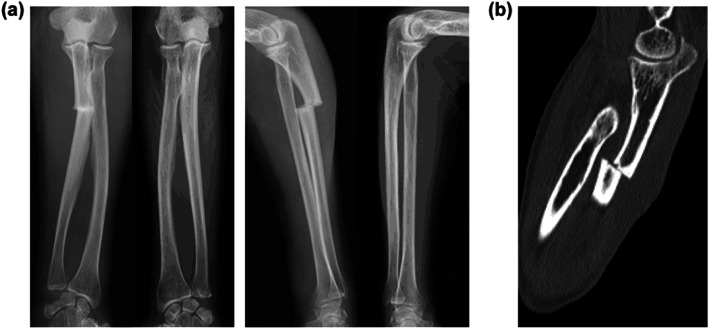
**a** Preoperative radiography of the bilateral forearm; **b** preoperative computed tomography. Sclerotic changes are seen at the fracture site

**Fig. 2:**
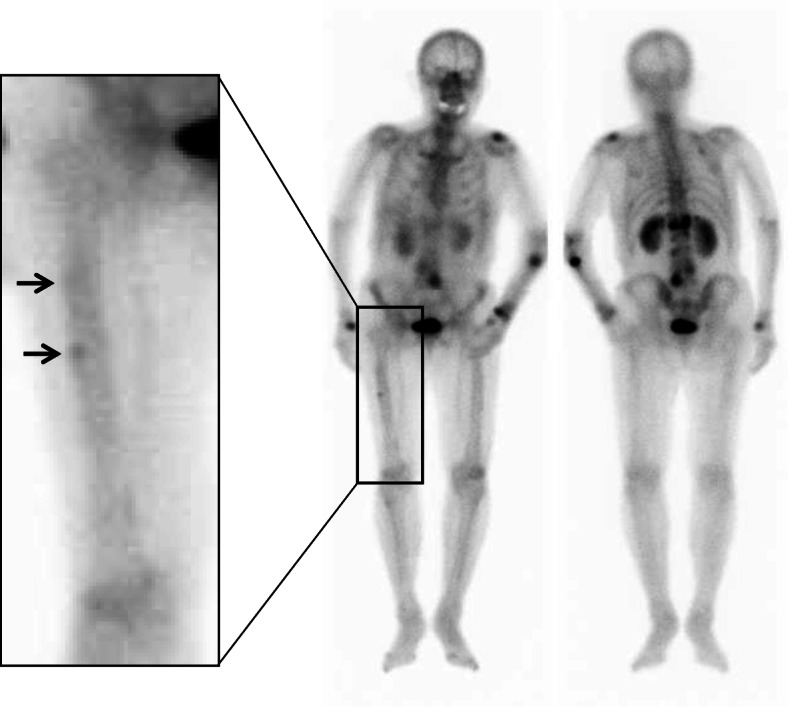
Whole body bone scan. The two arrows indicate abnormal tracer uptake

**Fig. 3:**
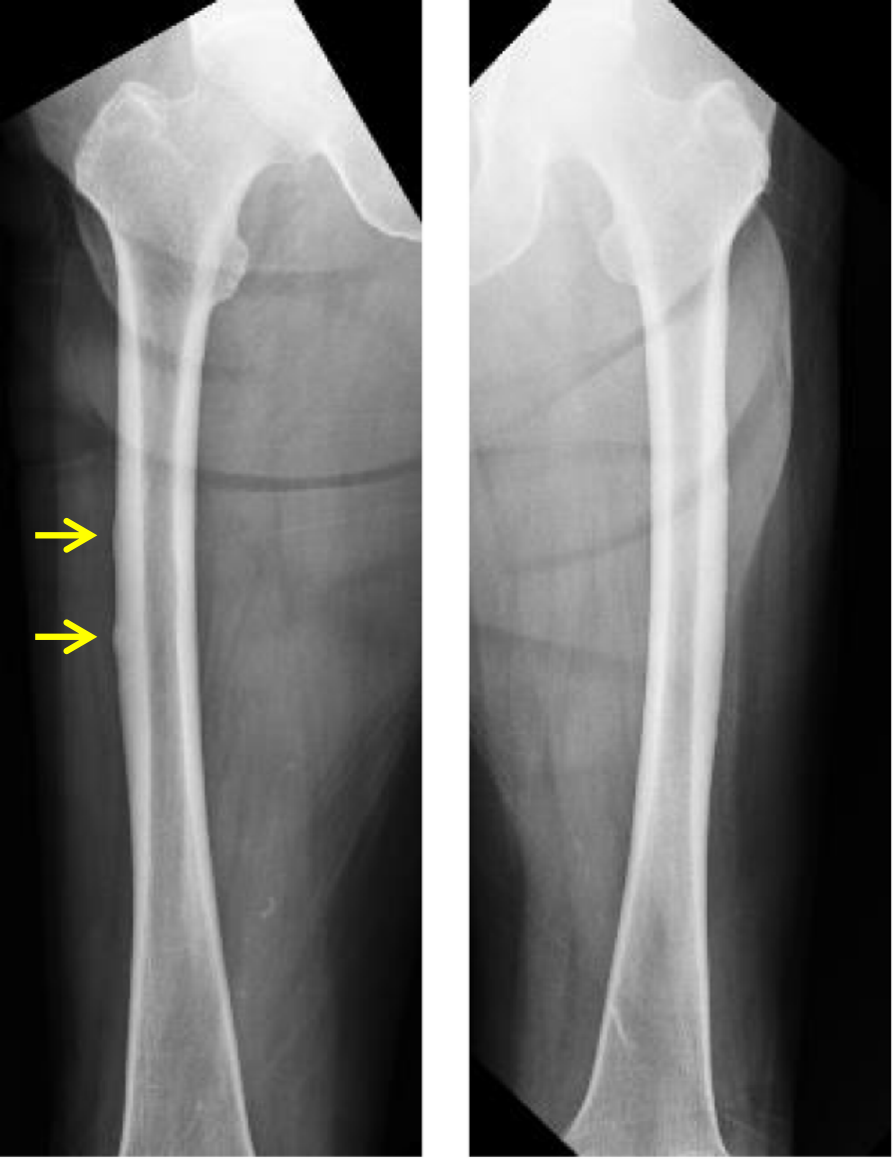
Radiography of the femur. The two yellow arrows indicate external bone reaction

An 84-year-old woman sustained a left ulnar shaft fracture due to fall. Prior to the fall, she had been aware of pain in her forearm while carrying a heavy luggage. She was on bisphosphonate therapy (ibandronate and alendronate) for more than 10 years; therefore, the fracture was considered an atypical fracture due to SSBT. Radiography and computed tomography showed a transverse fracture in the proximal third of the left ulnar shaft; in addition, there were sclerotic changes at the fracture site (Fig. 1). Among laboratory data, total type I procollagen-N-propeptide (bone formation marker) level was 15.5 ng/mL (reference range: 26.4–98.2) and tartrate-resistant acid phosphatase-5b (bone resorption marker) level was 138 mU/dL (120–420). These laboratory data indicated severe suppression of bone turnover. Bone mineral density of the L2–L4 was 0.974 g/cm^2^ (*t*-score: − 1.5), which is not in the osteoporotic range defined by the World Health Organization [23]. Whole body 99mTc-methylene diphosphonate bone scan showed abnormal tracer uptake not only at the fracture site but also at the right femur (Fig. 2). Radiography of the right femur also showed an external bone reaction (Fig. 3). Based on the patient work-up, two problems were identified. One was the atypical fracture of the left ulna, and the other was the external bone reaction of the right femur due to SSBT. Owing to the lack of any pain in the right thigh, no active treatment was planned for the femoral lesion apart from administration of teriparatide and close monitoring. Surgery was performed for the former condition. The surgical strategy is summarized below.

**Fig. 4:**
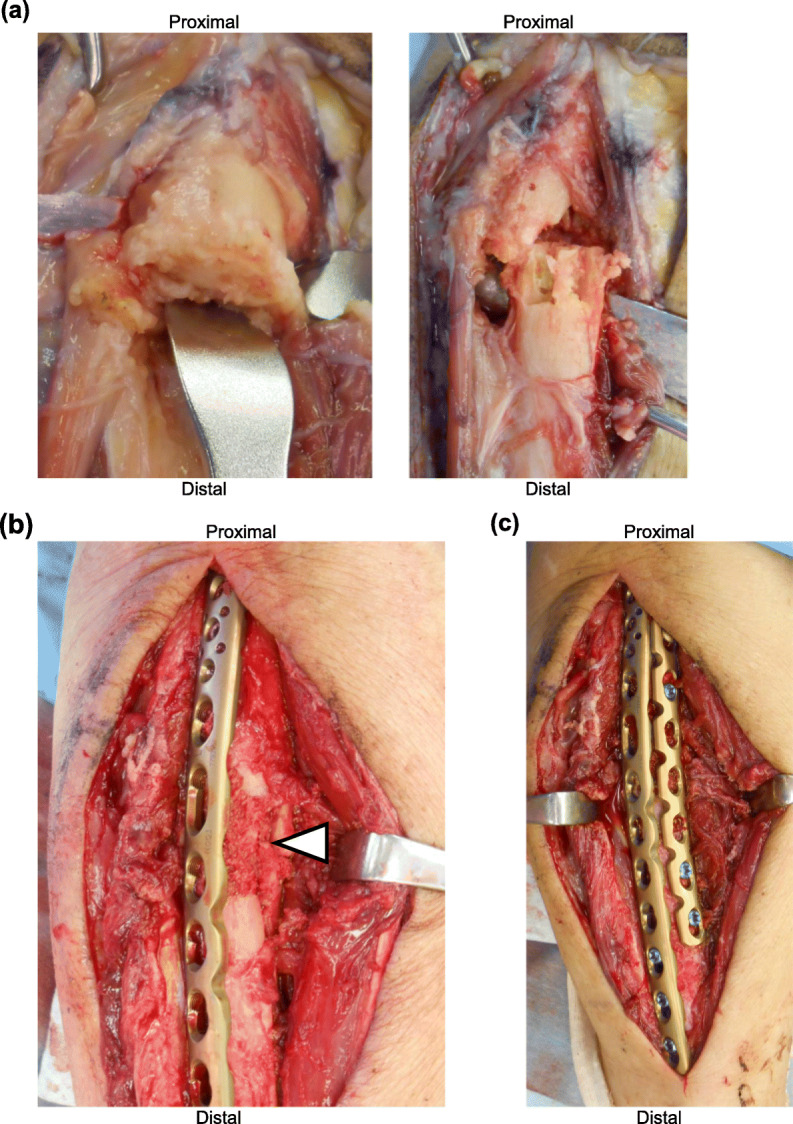
Operative photos. **a** Left panel shows cortical thickening at the fracture site. Right panel shows the surgical view after drilling and decortication. **b** White arrow head shows autogenous cancellous bone graft. **c** Internal fixation using dual plate
ii) Autogenous cancellous bone graft from iliac crest (Fig. 4b)
iii) Open reduction and internal fixation (ORIF) using dual plate (VA-LCP Proximal ulna plate and LCP-Reconstruction plate; DePuy Synthes, Zeist, Netherlands) (Fig. 4c)
iv) Low-intensity pulsed ultrasound (LIPUS) treatment (Accellus; NIPPON SIGMAX Co. Ltd., Tokyo, Japan)
v) Administration of teriparatide (56.5 μg/week: normal dose)

i) Drilling and decortication (Fig. 4a)

**Fig. 5:**
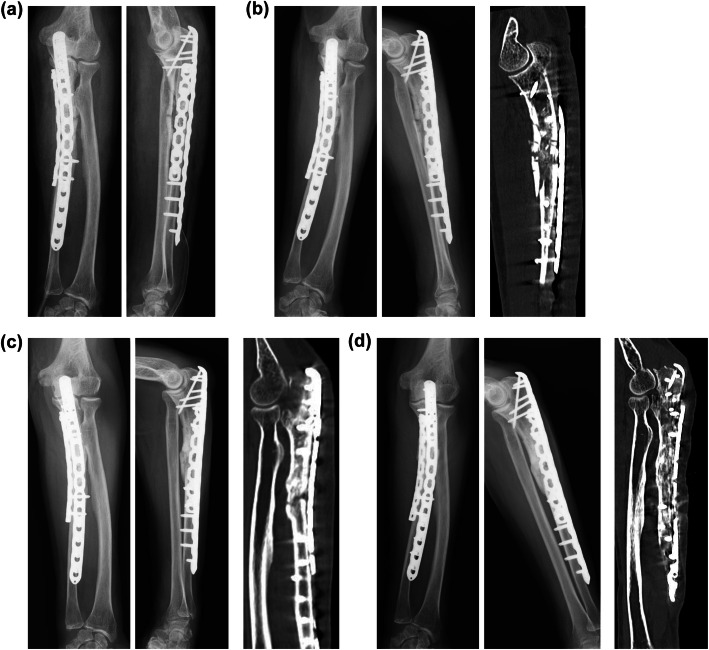
Postoperative radiography and computed tomography. **a** Post-operation (both panels, radiography). **b** 1 month after surgery (left two panels, radiography; right panel, computed tomography). **c** 6 months after surgery (left two panels, radiography; right panel, computed tomography). **d** 11 months after surgery (left two panels, radiography; right panel, computed tomography)

The transverse fracture with cortical thickening was evident during the procedure (Fig. 4a). Dual plate was used for ORIF in order to achieve rigid stability at the fracture site and to avoid implant failure due to the typically long time required for bone union. Permanent histologic section of the fracture site showed reactive bone formation, little osteoblast activation, and few osteoclasts, which was consistent with SSBT. The tendency for bone union was observed at 8 months after the operation; bone union was observed at 11 months (Fig. 5). The elbow and wrist joints showed almost complete range of movements with no pain. Moreover, bone mineral density of the L2–L4 improved to 1.050 g/cm^2^ (*t*-score: − 1.0).

## Discussion and conclusions

Atypical fracture of the ulna due to SSBT is a rare entity [6]; therefore, there is no standardized treatment strategy. In addition, widely variable treatment outcomes have been described in previous reports. The long time required for bone union is a major pit fall in the treatment of atypical ulnar fracture. This sometimes leads to non-union or implant failure. We conducted a literature review and identified 18 previous reports. Herein, we summarize these case reports and present our recommended treatment strategy.

Eight cases underwent conservative therapy; however, all cases resulted in non-union (follow-up duration: 6 weeks–1.5 years) [15–22]. Therefore, conservative therapy is unlikely to lead to bone union.

Nine cases underwent only ORIF using single plate; these included cases that underwent ORIF after failure of conservation therapy at first attempt. Of these, the treatment outcome was not reported for one case while two cases resulted in union (follow-up duration: unknown); however, other six cases (66.7%) resulted in non-union with or without implant failure (follow-up duration: 3 weeks–13 months). Two cases that showed non-union after ORIF alone underwent a second trial of ORIF alone; of these, one resulted in union at 1 year after surgery while another resulted in non-union and implant failure at 2 months after surgery [5, 6, 11–13, 19, 20]. In particular, the rate of implant failure was very high [41.7% (5/12) including the second and third treatment]. Therefore, although bone union may be achieved with ORIF alone using single plate, the success rate is quite low.

ORIF with additional treatment was reported in 15 cases (including second and third treatment). Additional treatment included use of bone graft, dual plate, LIPUS, and administration of teriparatide. The outcomes of additional treatment and mean duration of follow-up are summarized in Table 1 [6–14, 20–22]. Details of drilling and decortication are not included in Table 1 owing to the very few reports that described drilling and decortication. Bone graft seems to facilitate union; however, LIPUS and administration of teriparatide do not seem particularly useful for achieving union.

**Table 1:** Outcomes of additional treatment and the mean follow-up duration in previous reports

	Union (n)	Mean follow-up duration (months)	Non-union (n)	Mean follow-up duration (months)	Remarks
Bone graft	8	15.5^a^	2	6.5	^a^*n* = 6
Dual plate	2	6	0	N/A	
LIPUS	4	21	3	4.7	
Teriparatide	5	15.8^b^	3	4.7	^b^*n* = 4
Others	3	11.3	0	N/A	high-dose vitamin D3, high-dose teriparatide, cast immobilization

*LIPUS* Low-intensity pulsed ultra sound, *N/A* Not applicable

^a^ Mean value of the reported follow-up duration (not shown in two cases)

^b^ Mean value of the reported follow-up duration (not shown in one case)

Although there are few reports on surgical treatment for atypical fracture using bone grafts, both Ito et al. and Shimada et al. described successful treatment of atypical ulnar fractures by internal rigid fixation and use of bone graft. They resected the sclerotic region and inserted an autologous corticocancellous iliac bone graft [6, 9]. In our case, we performed drilling, decortication, and cancellous iliac bone grafting instead of the resection of the sclerotic region and autologous corticocancellous iliac bone grafting. However, the bone union was achieved. Therefore, refreshing the sclerotic site and use of bone graft may be important for achieving union.

Ito et al. also described that administration of teriparatide and LIPUS could be useful; however, there is no robust evidence of the efficacy of LIPUS in the treatment of atypical fractures [6]. According to Shimada et al., further investigations are required to determine whether the combination of teriparatide and LIPUS is effective in the treatment of atypical fractures [9]. The information presented in Table 1 does not support the usefulness of LIPUS and teriparatide therapy. However, owing to the extremely short duration of follow-up in the non-union group, no definitive conclusions can be drawn in this regard. Owing to the long time required for bone union in atypical fracture, longer follow-up is necessary to evaluate the usefulness of these treatments. Moreover, a previous report has described the usefulness of teriparatide in the treatment of atypical femoral fractures [24]. Yeh WL, et al. reported that the majority of patients (75%) treated with teriparatide treatment could achieve bone union within 6 months, with a mean time period of 4.4 months [25]. Moreover, a previous case report has described an additive effect of teriparatide and LIPUS during fracture healing [26]. Hence, LIPUS and teriparatide may be useful for achieving union, although further studies are required to obtain more definitive evidence.

Teriparatide is possibly effective for achieving bone union. Bisphosphonates are considered the first line of treatment for osteoporosis, and teriparatide is considered the second line. Use of bisphosphonates has been well reported to decrease the coincidence of hip fracture by 30% [27–29]. Edward et al. reported bisphosphonate-associated non-healing femoral fractures in the review of data from the US FDA Adverse Event Reporting System (1996–2011) and concluded that the benefits of bisphosphonate use are 100-fold greater than the risk of atypical femoral fractures [30]. However, the incidence is low [31], and drug holiday should be considered if the patient is below the moderate risk (*t*-score > − 2.5) group to avoid the risk of atypical fractures caused by long-term administration of bisphosphonates by the research of bisphosphonate-treated cases for 3–5 years [32]. In addition, serum osteocalcin level increased after approximately 3 months of teriparatide treatment [24]. As observed in the Sousa et al. study, the short-term increase in osteocalcin level was significant with the use of teriparatide in patients previously treated with bisphosphonates [33]. In the present case, bone formation marker levels were low and bone mineral density was at moderate risk. With the suspension of bisphosphonates and the initiation of teriparatide, bone mineral density tended to improve over the course of 1 year. If the patient had been examined for bone mineral density before this fracture, it could have been confirmed that she was at moderate risk, and as a result, it might have helped in deciding the suspension of bisphosphonate. Fractures may have been prevented because of the early administration of teriparatide with the aim of improving the low levels of bone formation markers. Romosozumab [34], which promotes bone formation, may be useful when teriparatide is not available. However, because the risk of atypical fracture is stated in the drug package insert (literature on romosozumab and atypical fracture was not found), further studies are required.

Some reports described that LIPUS was useful for bone unions in atypical femoral fractures (AFFs). Lee, et al. reported that AFFs that were not treated with LIPUS healed at 29 months after surgery, which was longer than the average time required for union in other AFFs that were treated with LIPUS. They described that LIPUS may be a potentially useful tool for accelerating AFF repair [35]. Of note, Arakawa, et al. suggested that for bisphosphonate-associated AFFs, LIPUS could be an alternative to parathyroid hormone analogs, such as teriparatide, that are contraindicated in patients with malignant skeletal metastases, and that LIPUS is an effective treatment for fracture healing [36].

Although there are few reports on ORIF using dual plate for atypical fractures, only two cases underwent ORIF with dual plate; however, four cases (16.7%) that underwent ORIF using single plate as the first treatment showed implant failure despite administration of additional treatments [11, 13, 14, 20]. This rate of implant failure is lower than that observed with only ORIF using single plate, but is still quite high. Okamoto et al. underlined the need for rigid internal fixation in the treatment of atypical fracture to avoid implant failure; they reported the usefulness of the dual plate for this purpose [11]. Indeed, ORIF using dual plate may help avoid implant failure.

Based on the above evidence, ORIF alone is not sufficient to achieve bone union; additional drilling, decortication, and cancellus bone graft (or resection of sclerotic site and corticocancellous bone graft) are essential to achieve favorable outcomes. Although further investigation is needed, administration of teriparatide and LIPUS may facilitate early bone union. Furthermore, ORIF using dual plate is considered necessary to avoid implant failure because of the long time required for bone union.

In our patient, we performed ORIF in combination with all additional treatments (drilling and decortication, bone graft, dual plate, LIPUS, and teriparatide) for atypical ulnar fracture. Although it required 11 months to achieve bone union, it was possible to achieve bone union without implant failure.

## Supplementary Information

**Additional file 1.** Summary of treatment strategies for atypical ulnar fracture and outcomes in previous reports. This table summarizes the treatment strategies and outcomes of atypical ulnar fracture in 25 cases described in 18 previous reports and the present case.

## Data Availability

To protect privacy and respect confidentiality, no raw data have been made available in any public repository. The original operation reports, intraoperative photographs, imaging studies, and outpatient clinic records are retained as per the normal procedure within medical records of our institution. The datasets used and/or analyzed during the current study available from the corresponding author on reasonable request.

## Abbreviations

SSBT: Severely suppressed bone turnover
ORIF: Open reduction and internal fixation
LIPUS: Low-intensity pulsed ultrasound
AFF: Atypical femoral fracture
