# Correction to: Treatment strategy for atypical ulnar fracture due to severely suppressed bone turnover caused by long-term bisphosphonate therapy: a case report and literature review

**DOI:** 10.1186/s12891-020-03908-9

**Published:** 2021-01-05

**Authors:** Kensaku Abe, Hiroaki Kimura, Norio Yamamoto, Shingo Shimozaki, Takashi Higuchi, Yuta Taniguchi, Takaaki Uto, Hiroyuki Tsuchiya

**Affiliations:** 1 Department of Orthopaedic Surgery, Japanese Red Cross Kanazawa Hospital, 2-251 Minma, Kanazawa, 921-8162 Japan; 2 Department of Orthopaedic Surgery, Graduate School of Medical Sciences, Kanazawa University, 13-1 Takara-machi, Kanazawa, 920-8641 Japan

**Correction to: BMC Musculoskelet Disord 21, 802 (2020)**

**https://doi.org/10.1186/s12891-020-03824-y**

Following publication of the original article [1], the authors noticed that the corresponding author was published incorrectly. The correct corresponding author is Dr. Hiroaki Kimura.

The original article [1] has been updated.
